# A continuous-time hidden Markov model for cancer surveillance using serum biomarkers with application to hepatocellular carcinoma

**DOI:** 10.1007/s40300-019-00151-8

**Published:** 2019-05-30

**Authors:** Ruben Amoros, Ruth King, Hidenori Toyoda, Takashi Kumada, Philip J. Johnson, Thomas G. Bird

**Affiliations:** 1 School of Mathematics, University of Edinburgh, Edinburgh, EH9 3FD UK; 2 Department of Gastroenterology, Ogaki Municipal Hospital, Ogaki, Japan; 3 Institute of Translational Medicine, University of Liverpool, Liverpool, UK; 4 Cancer Research UK Beatson Institute, Switchback Road, Glasgow, G61 1BD UK; 5 MRC Centre for Inflammation Research, The Queens Medical Research Institute, University of Edinburgh, Edinburgh, EH16 4TJ UK

**Keywords:** Hidden Markov chains, Hepatocellular carcinoma, Disease detection, Change-point models

## Abstract

Hepatocellular carcinoma (HCC) is the fourth most common cause of cancer deaths worldwide, and its early detection is a critical determinant of whether curative treatment is achievable. Early stage HCC is typically asymptomatic. Thus, screening programmes are used for cancer detection in patients at risk of tumour development. Radiological screening methods are limited by imperfect data, cost and associated risks, and additionally are unable to detect lesions until they have grown to a certain size. Therefore, some screening programmes use additional blood/serum biomarkers to help identify individuals in whom to target diagnostic cancer investigations. The GALAD score, combining the levels of several blood biomarkers, age and sex, has been developed to identify patients with early HCC. Here we propose a Bayesian hierarchical model for an individual’s longitudinal GALAD scores whilst in HCC surveillance to identify potentially significant changes in the trend of the GALAD score, indicating the development of HCC, aiming to improve early detection compared to standard methods. An absorbent two-state continuous-time hidden Markov model is developed for the individual level longitudinal data where the states correspond to the presence/absence of HCC. The model is additionally informed by the information on the diagnosis by standard clinical practice, taking into account that HCC can be present before the actual diagnosis so that there may be false negatives within the diagnosis data. We fit the model to a Japanese cohort of patients undergoing HCC surveillance and show that the detection capability of this proposal is greater than using a fixed cut-point.

## Introduction

Primary liver cancer, of which hepatocellular carcinoma (HCC) is the most common form, is the fourth highest cause of cancer deaths worldwide, accounting for 840,000 cases and 780,000 deaths annually with an age adjusted incidence of 9.5 case per 100,000 person years [5]. The development of HCC is closely linked to the presence of underlying chronic liver disease. Despite there being global prevention strategies, such as targeting Hepatitis B and C through vaccination and treatment respectively [11], at present the incidence of HCC in both low and high income countries is rising and outcomes for patients with HCC remain typically among the worst of any cancer type [5]. HCC stage at diagnosis is the major determinant of the treatment that can be applied and whether curative treatment is achievable. Thus, early stage HCC detection is essential to improve outcomes for individuals who develop HCC. Achieving detection of HCC at an early stage is challenging as the disease is typically asymptomatic. Therefore, cancer surveillance strategies are required. Due to the associated cost and practicality constraints, these programmes are generally targeted to those at highest HCC risk, with stratification revolving centrally around the degree of liver fibrosis. Cirrhosis, the end-stage of liver fibrosis, is the highest risk factor associated with HCC, with approximately 70–90% of HCC cases having established cirrhosis [8]. The incidence of HCC among persons with cirrhosis is also high, with an estimated 1 in 3 people with cirrhosis eventually developing HCC. Thus, significant underlying liver disease is typically used as an entry point into HCC surveillance programmes. These surveillance programmes usually rely upon imaging based HCC detection, typically using ultrasound, which may also be supplemented by serum biomarkers [12, 14, 20]. The actual diagnosis of HCC, following suspicion of underlying tumour raised by screening, is achieved by diagnostic cross sectional imaging; multiphasic computed tomography and/or dynamic contrast-enhanced magnetic resonance imaging. Each of these radiological tests has their own limitations including expense, radiation exposure, diagnostic accuracy—particularly in the presence of cirrhosis or presence of fat in the liver—and an inability to accurately diagnose small lesions ($<1$ cm). These tests may, however, be supplemented with the current gold standard of histopathological examination following a targeted liver biopsy, an invasive procedure itself carrying risk. An accepted means of identifying screened individuals for diagnostic investigation is to use blood/serum biomarkers either in isolation or jointly with image-based surveillance programmes. The measurement of biomarkers using simple blood tests is less expensive than radiological imaging tests and has the potential of detecting the development of tumours before they may be confirming on imaging [17].

Levels of several blood biomarkers, including Alpha-fetoprotein (AFP), des-carboxy-protrombin (DCP) and AFP-L3 (an isoform of AFP) have been shown to be indicative of the development of HCC and are included in specific international guidelines [21]. The GALAD score, defined to be a linear combination of the concentration of these three biomarkers (log transformed for AFP and DCP) combined with age and gender, has been proposed for distinguishing patients in HCC surveillance having underlying tumours. In the original cross-sectional study by Johnson et al. [17], sensitivities of over 97% and specificities of over 62% were reported using a (fixed) threshold score as the criterion. The construction of the threshold score was based on a logistic regression analysis of data from a cross-sectional study and classifies a patient as having a high risk of developing HCC if their GALAD score exceeds a given threshold. This approach was subsequently validated in cross sectional analysis of international cohorts [3]. However, this does not necessarily imply that a fixed threshold for the GALAD score (or any of the individual biomarkers) will be as effective in detecting the start or growth of the tumour, nor that alternative strategies for detecting changes in biomarker behaviour could not provide improved tumour detection accuracy. Observational longitudinal data of individual biomarker levels (AFP) suggest that many patients who later go on to be diagnosed as developing HCC show a gradual (approximately log-linear) increase of their AFP biomarker level before being diagnosed with HCC [4].

Using the observational data and expert medical knowledge, several approaches have been taken to build models that take into account the temporal evolution of the biomarker or GALAD score per patient. These include, for example, a parametric empirical Bayes approach that weights the population threshold with the historic observations of the patients at risk of ovarian cancer [10, 23] and HCC [33]; or joint longitudinal-survival models for prostate cancer [28, 36]; and modelling the transition probabilities from cirrhosis to HCC and/or death via longitudinal hidden Markov models (HMMs; [2]). Here, we focus on directly modelling the individual longitudinal GALAD scores of patients in HCC surveillance. To study this we use a Bayesian hierarchical model to permit the identification of the potential change in trend of the score (from no temporal trend to an increasing trend) corresponding to the onset of a tumour. In particular, we develop an absorbent continuous-time HMM with two states, corresponding to “tumour free” (or HCC absent) or “tumour present” (or HCC present) with an associated observation process corresponding to the GALAD score with constant (but individual-specific) mean for the state of tumour free and a linearly increasing mean for the state of tumour present (from the onset of the tumour). Further, the transition probability of moving from a tumour free state to the tumour present state (i.e. developing HCC) is also increased with higher patient baseline GALAD levels. We have additional observational data relating to the diagnosis of patients. The diagnosis of HCC is specified as a partial observation of the underlying latent variable (presence versus absence of HCC) where we assume that there are no false positive diagnoses (all individuals diagnosed with HCC do have a tumour) but where there may be false negatives (an individual may not yet be diagnosed with HCC but may have an underlying undiagnosed tumour). We note that permitting false negatives means that the lack of a correct positive diagnosis (e.g. when the tumour is too small to identify using image-based diagnostic techniques) does not lead to biased parameter estimation due to this incorrectly assumed state of the individual. Finally, the proposed model incorporates a mixture component which allows for a proportion of the patients that develop HCC to not display any change in their AFP or GALAD score over time [3, 4]. We note that this final issue implies that sensitivity based on the use of such biomarkers will always be imperfect, but we are able to estimate the proportion of individuals for whom such biomarker analyses will not provide an early indicator of HCC, for the given representative population.

Similar modelling approaches have been proposed and applied to ovarian cancer [22, 30] and HCC [34], including the use of several biomarkers simultaneously. However, these previous approaches have further limitations, such as the necessity for the training of the models to distinguish patients with and without cancer without error; or not considering the individual heterogeneity of the baseline biomarker level of each patient as a risk factor for developing a tumour. Further, the time of developing HCC is modelled with respect to the time of diagnosis, and so is specified retrospectively. Conversely in our approach we more intuitively model the time until diagnosis, given the time of developing HCC, so that prediction of the presence of HCC can be made without knowing a time of diagnosis (never known in prospective detection).

The rest of the paper is structured as follows. In Sect. 2 we detail the patient dataset we use in this work; Sect. 3 presents the proposed statistical model in detail; the results of applying this model to our motivating dataset are presented in Sect. 4; and conclusions are given in Sect. 5.

## Patient data

We focus on the dataset provided by the Ogaki Municipal Hospital, Japan, comprising of individual longitudinal data collected at irregular times over the period of several years from patients with cirrhosis being screened for HCC. In total, data from 2273 patients were available between the years 2009–2015. We discarded one patient record as no GALAD scores were recorded; of the remaining 2272 patients, 113 (5%) were diagnosed with HCC within the study period. These data provided a total of 50,410 observations. Of these observations, 1807 corresponded to future GALAD scores after a patient is diagnosed with HCC and a further 13,805 observations failed to record a complete GALAD score, so that these observations were omitted from the data—though we retained 93 observations corresponding to HCC diagnosis dates that where kept in the database even though the GALAD score was not recorded at the time—leaving a total of 35,001 observations (or a mean of approximately 15 observations per patient). In addition to the GALAD scores, additional covariates recorded on each patient included sex, cirrhosis aetiology, vital status, age, date of observations and, for those diagnosed with HCC, the date of HCC diagnosis, number of tumours and maximum size of the tumours. Table 1 offers a description of these variables segmented by HCC-diagnosed and non-diagnosed patients. The dataset was randomly divided into a training dataset and a test dataset comprising 75% and 25% of the patients respectively stratified by HCC diagnosis (26,356 and 8645 observations respectively). The distribution of the rest of the covariates was checked and found to be similar in both training and test datasets. Importantly, in order to minimize the possibility of false negatives from the earliest forms of HCC in the test dataset, 3380 observations were excluded, corresponding to all the observations from non-diagnosed patients that were less than two years before their last observation of the study. This two year period was chosen based upon a realistic timescale for the undiagnosed tumours to make themselves apparent, taking into account both tumour doubling time and survival following early diagnosis.

**Table 1:** Description of the database provided from the Ogaki Municipal Hospital, Japan, stratified by diagnosed and non-diagnosed with HCC patients

	Diagnosed		Non-diagnosed	
Sex				
Female	36 (31.9%)		1153 (53.4%)	
Male	77 (68.1%)		1006 (44.6%)	
Aetiology				
HBV	15 (13.3%)		581 (26.9%)	
HCV	83 (73.4%)		1078 (49.9%)	
HC+BV	1 (0.9%)		40 (1.9%)	
Other	14 (12.4%)		460 (21.3%)	
Vital status				
Alive	94 (83.2%)		2121 (98.2%)	
Dead	19 (16.8%)		38 (1.8%)	
Age (years)	68.1 (9.2)	34.1 $\rightarrow$ 85.0	61.6 (13.2)	11.7 $\rightarrow$ 92.2
Obs. per patient	13.5 (7.0)	2 $\rightarrow$ 39	15.5 (6.4)	6 $\rightarrow$ 70
GALAD score	$-$ 1.08 (2.0)	$-$ 6.1 $\rightarrow$ 12.4	$-$3.1 (1.9)	$-$9.5 $\rightarrow$ 7.8
Screening time (years)	3.2 (1.2)	1.0 $\rightarrow$ 5.6	5.1 (0.7)	3.0 $\rightarrow$ 6.0
Num. tumours				
1	85 (75.2%)			
2	22 (19.5%)			
3	4 (3.5%)			
5	2 (1.8%)			
Max. size tumours (cm)	2.1 (1.0),	0.7 $\rightarrow$ 7.6		
Total patients	113 (100%)		2159 (100%)	

Number of patients (percentage%) for discrete variables. Mean (SD), minimum $\rightarrow$ maximum for continuous variables. *Obs*. observations, *Num*. number, *Max*. maximum, *HBV* hepatitis B virus, *HCV* hepatitis C virus, *HC*$+$*BV* both hepatitis C and B virus

## Method

In this section we initially present the notation that we shall use throughout before constructing the hidden Markov model motivated by the current clinical understanding of HCC.

### Notation

We let *I* denote the total number of patients, and index each patient by *i* in $1, \ldots , I$. We let $J_i$ denote the number of observations recorded for patient $i=1,\ldots ,I$, and index each chronologically ordered observation for patient *i* by $j=1,\ldots ,J_i$ (observation occasion). Finally we let $t_{ij}$ denote the time (in days) from the first screening of patient *i* to observation *j*; so that $t_{i1} = 0$ for all $i=1,\ldots ,I$. The observed data correspond to the set of observed GALAD scores for each patient $i=1,\ldots ,I$ denoted by ${\mathbf {B}}_i = \{B_{ij}:j=1,\ldots ,J_i\}$ and for those individuals who are diagnosed with HCC, the associated time of diagnosis. Consequently, from the date of positive diagnosis (if any) we obtain the associated diagnosis status denoted by the indicator function $D_{ij}$ corresponding to whether or not individual $i=1,\ldots ,I$ has been diagnosed with HCC at observation occasion $j=1,\ldots ,J_i$; and let all of diagnosis states for individual *i* be denoted by ${\mathbf {D}}_i = \{D_{ij}:j=1,\ldots ,J_i\}$. Thus we can express the data as the set of paired observations of GALAD score and diagnosis status of each patient at each observation occasion, $\{B_{ij}, D_{ij}: i=1,\ldots ,I; j=1,\ldots ,J_i\}$ which we combine with the associated known set of screening times for each patient, $\{t_{ij}: i=1,\ldots ,I; j=1,\ldots ,J_i\}$.

### Model

**Fig. 1:**
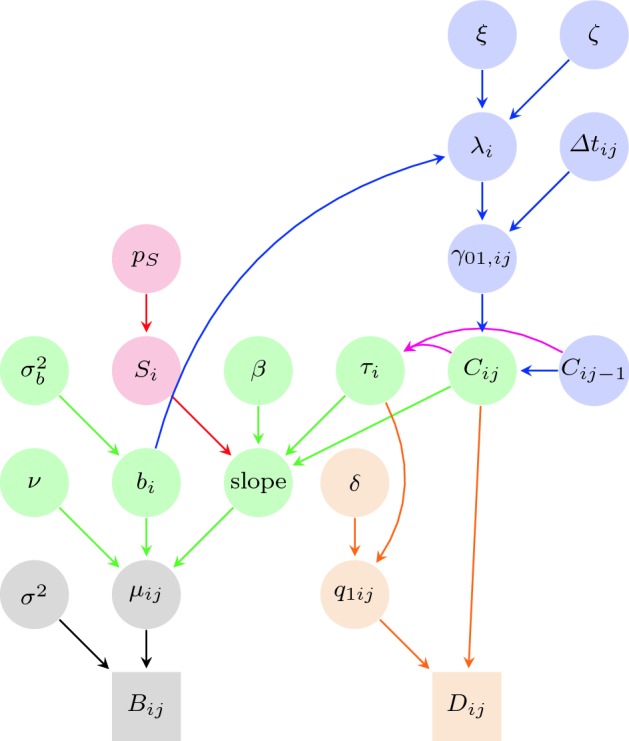
Directed graph representing the hierarchical model. The grey components correspond to the conditional distribution for the observed GALAD scores; the green components to the specification of the mean underlying GALAD score over time; the red components to the mixture distribution to account for the behaviour of biomarkers given a tumour present; the blue components to the underlying latent process of disease status; and the orange components to represent the diagnosis observation, given the disease status of an individual (colour figure online)

We develop a continuous-time hidden Markov model to represent the processes that are acting on the system. The overall model is summarised in Fig. 1 in the form of a directed graph, providing the direct conditional relationships between the model parameters. We describe each of the components of the hierarchical model in turn.

#### Observed GALAD score

For each patient $i=1,\ldots ,I$ and observation occasion $j=1,\ldots ,J_i$, the GALAD score $B_{ij}$ (or more generally a biomarker score) is recorded. We assume that all the recorded GALAD scores are conditionally independent observations from a Gaussian distribution with mean $\mu _{ij}$ and variance $\sigma ^2$, such that,

1 $$\begin{aligned} B_{ij} | \mu _{ij}, \sigma ^2 \sim {{\,\mathrm{{\mathcal {N}}}\,}}(\mu _{ij}, \sigma ^2), \end{aligned}$$

where $\mu _{ij}$ is itself a function dependent on numerous factors, but critically on whether an individual has a tumour or not—we discuss this mean in detail in Sect. 3.2.2. Component (1) of the model is represented in grey in Fig. 1, and describes the first level of the hierarchical model. We note that for our data there are 93 observation corresponding to a confirmed diagnosis for which there is no associated GALAD score recorded, in this case we simply omit these GALAD scores in this part of the model. In general, alternative distributions may be appropriate for other biomarker data, such as raw AFP values which are constrained to be positive so that a log-Normal distribution may be considered.

#### Underlying mean GALAD level

Here we consider how the underlying mean GALAD score of an individual, $\mu _{ij}$, may change over time—and consider two separate cases: (i) individuals that do not develop HCC within the study; and (ii) individuals who do develop HCC within the study and describe each in turn.

#### Case (i): non-HCC patients

We assume that a patient without HCC has a constant underlying mean GALAD score. However, this baseline score will be different for each patient, and therefore can be expressed as the sum of a population mean baseline, denoted by $\nu$, and an individual heterogeneity term, $b_i$, modeled as a Gaussian random effect.

#### Case (ii): HCC-positive patients

We initially assume that individuals who develop HCC will experience an increase in their underlying mean GALAD score. We note that this assumption is not always true, but for simplicity we omit this complexity at this stage and we extend the model to account for this below. We let $\tau _i$ denote the (unobserved) time when a tumour starts to grow for individual *i*, given that they develop HCC within the study, and assume that this coincides with the change in the mean underlying GALAD score. If there is a lag between a tumour developing and a change in GALAD score then the definition of $\tau _i$ changes accordingly to be the time at which the mean GALAD score changes (driven by changes in the biomarkers) following the development of the tumour. Prior to the change-point $\tau _i$ the mean GALAD score for an individual is as for non-HCC patients. Following the onset of the tumour, we assume that underlying mean GALAD score increases linearly over time.

Mathematically combining the two cases together we can express the above in the form:

2 $$\begin{aligned} \mu _{ij}&= \nu + b_i + C_{ij} \beta (t_{ij} - \tau _i)\,, \nonumber \\&b_i|\sigma _b^2 \sim {{\,\mathrm{{\mathcal {N}}}\,}}(0, \sigma _b^2)\,, \end{aligned}$$

such that $C_{ij} \equiv I(\tau _i < t_{ij})$, where $I(\cdot )$ denotes the indicator function and we use the convention that $\tau _i = \infty$ for individuals who do not develop a tumour within the study period. In other words, $C_{ij} = 0$ if the patient does not have a tumour at time $t_{ij}$; and $C_{ij} = 1$ if the patient does have a tumour at time $t_{ij}$. Finally, we note that we constrain the slope parameter $\beta > 0$ for clinical reasons. This aspect of the model is denoted by the green components of Fig. 1.

However, as noted above, not all individuals who develop HCC have the same biological reactions. In particular, there appears to be a proportion of HCC-positive individuals for whom there is no discernible change in their GALAD score prior to diagnosis. To account for this mathematically we consider a mixture model where there are two homogeneous sub-populations. Sub-population (or mixture component) 1 corresponds to those whose mean GALAD scores do increase following the onset of HCC (linearly in time as described above); and sub-population (or mixture component) 2 to those for whom their mean underlying GALAD score is unchanged despite the development of a tumour. We let $p_S$ denote the probability that a patient who develops HCC will have an increased mean GALAD score (i.e. belongs to sub-population 1). This additional mixture model is represented in Figure 1 by the red components.

The underlying mean GALAD score can then be written as a mixture distribution. However, we will consider a (Bayesian) data augmentation approach, and introduce the additional indicator variable $S_i$ such that $S_i = 1$ if individual *i* belongs to sub-population 1 (for which there is a change in their mean underlying GALAD score if a tumour develops); and $S_i = 0$ if individual *i* belongs to sub-population 2 (for which there is no change in their mean underlying GALAD score if a tumour develops). We specify,

3 $$\begin{aligned} S_i|p_S&\sim {{\,\mathrm{Bernoulli}\,}}(p_S). \end{aligned}$$

For mathematical convenience we set ${\mathbf {S}} = \{S_i:i=1,\ldots ,I\}$. Further we can extend the above mathematical expression for the underlying mean GALAD score to account for the heterogeneity due to the two different sub-populations, specifying,

4 $$\begin{aligned} \mu _{ij}&= \nu + b_i + C_{ij} S_i \beta (t_{ij} - \tau _i)\,, \nonumber \\ b_i|\sigma _b^2&\sim {{\,\mathrm{{\mathcal {N}}}\,}}(0, \sigma _b^2). \end{aligned}$$

#### True underlying HCC state

In the above model specification the set of all variables ${\mathbf {C}} = \{C_{ij}: j=1,\ldots ,J_i; i=1,\ldots ,I\}$ determines the underlying disease status of the individuals over time. For notational purposes we also define ${\mathbf {C}}_i = \{C_{ij}: j=1,\ldots ,J_i\}$ corresponding to the disease status of individual *i* within the study period at the observation occasions. These variables are the latent states of the process (although we note that they are actually partially observed since we assume that there are no incorrect positive diagnoses—see Sect. 3.2.4). We assume a first-order Markov process for this latent process defined through the associated transition matrix, which defines the probability of the state (value) of the hidden variable at observation occasion $j+1$, denoted, $C_{ij+1}$ given the current state $C_{ij}$ at observation occasion *j*. This HMM is represented by the blue components in Fig. 1. In our case there are two possible states, 0 corresponding to the individual without HCC and 1 corresponding to an individual with HCC. The transition matrix, for individual *i* at observation occasion *j*, denoted $\varGamma _{ij}$, can be expressed in the form:

5 $$\begin{aligned} \varGamma _{ij}&= \left[ \begin{array}{cc} \gamma _{00,ij} &{} \quad \gamma _{01,ij} \\ \\ \gamma _{10,ij} &{} \quad \gamma _{11,ij} \end{array} \right] \,, \end{aligned}$$

with $\gamma _{ab,ij}={\mathbb {P}}(C_{ij+1}=b \, | \, C{ij}=a)$ for $a,b = 0,1$. Thus $\gamma _{01,ij}$ denotes the probability of developing HCC between the observation occasions *j* and $j+1$. HCC is almost universally a progressive disease and thus we assume that once a tumour has developed, it remains until it is diagnosed. In other words, state 1 (tumour present) is an absorbing state, so that, $\gamma _{11,ij}=1$ and conversely, $\gamma _{10,ij}=0$, for all $i=1,\ldots ,I$ and $j=1,\ldots ,J_i$.

Contiguous observations are not equidistant, so that the probability of developing cancer at observation $j+1$ should be dependent on the elapsed time since the previous observation, *j*, that is, dependent on $\varDelta t_{ij+1} = t_{ij+1} - t_{ij}$. Therefore, we consider a continuous-time transition matrix [9, 15] to model the development of HCC. In this case, the transition matrix $\varGamma _{ij}$ take the following form:

6 $$\begin{aligned} \varGamma _{ij}&= \exp (\varDelta t_{ij+1} \times \varLambda _{ij}) \,, \end{aligned}$$

where,

$$\begin{aligned} \varLambda _{ij}&= \left[ \begin{array}{cc} -\lambda _i &{} \quad \lambda _i \\ \\ 0 &{} \quad 0 \end{array} \right] \,, \end{aligned}$$

such that $\lambda _i >0$. We note that the second row of $\varLambda _{ij}$ is set equal to zeros because we assume that the HCC present state is an absorbing state.

We can explicitly express the associated transition matrix by computing the matrix exponential:

7 $$\begin{aligned} \varGamma _{ij}&= \left[ \begin{array}{cc} e^{-\lambda _i \varDelta t_{ij+1}} \quad &{} 1 - e^{-\lambda _i \varDelta t_{ij+1}} \\ \\ 0 &{} 1 \end{array} \right] \,. \end{aligned}$$

We note that this part of the model takes the form of a survival model with instant hazard function $\lambda _{i}$ for the time until the development of HCC, with the peculiarity that this event is not observed but is a hidden variable in our model. Previous studies have suggested that patients with higher levels of the biomarker AFP or GALAD scores are more prone to develop cancer [6, 17, 32]. To incorporate this within our model we specify the hazard rate $\lambda _i$ to be a log-linear function of the (instantaneous) mean individual random effect GALAD score component, $b_i$, such that:

8 $$\begin{aligned} \log (\lambda _i)&= \zeta + \xi b_i \,, \end{aligned}$$

where $\zeta$ and $\xi$ are parameters to be estimated. Thus the individual random effect component contributes to both the underlying mean GALAD score and acts as a risk factor for developing HCC within the model.

Now, if patient *i* develops HCC during the screening programmes, the time that the HCC develops (recall this is denoted by $\tau _i$) will be located between the time of the last observation when the patient was tumour free, denoted $t0_i$, and the time of the following observation when the patient has developed a tumour, denoted $t1_i$. We assume a conditional uniform prior distribution for the time of development of HCC, $\tau _i$, for the interval between consecutive screening times in which the tumour is developed (though not necessarily confirmed via diagnosis):

9 $$\begin{aligned} \tau _i&\sim {{\,\mathrm{U}\,}}(t0_i,t1_i) , \nonumber \\ t0_i&= t_{ij} \quad \text{ such } \text{ that } C_{ij} = 0 \hbox { and } C_{ij+1} = 1 ,\nonumber \\ t1_i&= t_{ij+1} \quad \text{ such } \text{ that } C_{ij} = 0\hbox { and } C_{ij+1} = 1. \end{aligned}$$

These dependencies are represented with magenta arrows in Fig. 1. Note that if a patient has HCC at their first observation time $t_{i1}$ (a very unlikely situation in our database), we assume that the time of development of the tumour is equal to $t_{i1} = 0$.

Finally, we note that within our model, the time of the development of HCC is conditionally dependent on the underlying HCC state of the individual and not on the time of diagnosis as is the case of other models previously proposed [30, 34]. This allows us to model this time realistically in prospective screening for future patients, where the information about a possible future diagnosis time will, by definition, be absent.

#### Diagnosis

A positive HCC diagnosis confirmed through the standard clinical diagnostic procedures (either imaging or biopsy) is only feasible after several months after the establishment of the first tumoural cell clones based upon tumour doubling times [1, 24, 29]. Consequently, the greater the time elapsed since the onset of the tumour, the longer the tumour has had to grow and, therefore, the larger it should be and the easier to detect. Therefore, the probability of a patient being diagnosed by standard clinical procedures can be modelled in the fashion of a survival analysis by means of the instant hazard of being diagnosed $\delta$ and the time elapsed since the development of HCC ($t_{ij} - \tau _i$).

Recall that the observed variable diagnosis, $D_{ij}$, takes the value 1 if patient *i* has been diagnosed at observation *j* and 0 otherwise, modelled with a Bernoulli distribution with parameter $q_{C_{ij}, ij}$:

$$\begin{aligned} D_{ij} | q_{C_{ij}, ij}&\sim {{\,\mathrm{Bernoulli}\,}}(q_{C_{ij}, ij}) \,, \end{aligned}$$

such that,

10 $$\begin{aligned} q_{1, ij}&= 1-e^{-\delta (t_{ij} - \tau _i)} \,, \nonumber \\ q_{0, ij}&= 0 \,. \end{aligned}$$

Defining $q_{0, ij} = 0$ ensures that if a patient has no HCC at observation *j*, they cannot be diagnosed, i.e. there are no false positives.

This way of modelling the diagnosis (represented in Fig. 1 in orange) allows the information of the diagnosis to inform the values of the variable $C_{ij}$ (and the rest of the parameters) by means of the inverse conditional relation between the two sets of variables. This has two distinct advantages. The first is that this modelling assumes the possibility for a patient in the training dataset to have HCC but have not yet been diagnosed. The second is that this part of the model can be ignored for the prediction (where diagnosis is always negative), as $\tau _i$ (the time of HCC development) is not directly dependent on any diagnosis—in other words the time of HCC development is conditionally independent of the diagnosis given the latent states ${\mathbf {C}} = \{C_{ij}:i=1,\ldots ,I, j=1,\ldots ,J_i\}$.

### Prior distributions for the hyperparameters

The model specification is completed by the prior distributions specified on the model parameters:

11 $$\begin{aligned} \beta&\sim {{\,\mathrm{U}\,}}(0, h_\beta ),&\nu&\sim {{\,\mathrm{{\mathcal {N}}}\,}}(0, h_\nu ),&\delta&\sim {{\,\mathrm{U}\,}}(0, h_\delta ), \nonumber \\ \sigma&\sim {{\,\mathrm{U}\,}}(0, h_\sigma ),&\zeta&\sim {{\,\mathrm{U}\,}}(h_{\zeta 1}, h_{\zeta 2}),&p_s&\sim {{\,\mathrm{Beta}\,}}(h_{S 1}, h_{S 2}), \nonumber \\ \sigma _b&\sim {{\,\mathrm{U}\,}}(0, h_{\sigma _b}),&\xi&\sim {{\,\mathrm{U}\,}}(h_{\xi 1}, h_{\xi 2}),&\end{aligned}$$

where $h_\beta , h_\sigma , h_{\sigma _b}, h_\nu , h_{\zeta 1}, h_{\zeta 2}, h_{\xi 1}, h_{\xi 2}, h_\delta , h_{S 1}$ and $h_{S 2}$, are defined such that they reflect the prior information available concerning the parameters or be chosen to express vague prior beliefs.

### Joint posterior distribution

We consider a Bayesian (data augmentation) approach to fit the model to the observed data corresponding to longitudinal GALAD scores, ${\mathbf {B}}$, and diagnosis output, ${\mathbf {D}}$. The associated model parameters are given by ${\varvec{\theta }}= \{\beta , \sigma ^2, \sigma ^2_b, \nu , \zeta , \xi , \delta , p_S\}$. We introduce the auxiliary variables, denoted ${\varvec{\phi }}= \{{\mathbf {C}}, {\varvec{\tau }}, {\mathbf {S}}, \mathbf{b }\}$, corresponding to the unobserved (and hence unknown) system state variables of the true HCC state for all individuals over screening times (${\mathbf {C}}$); the time of onset of HCC (if any) for all individuals (${\varvec{\tau }}$); sub-population mixture components (${\mathbf {S}}$); and associated underlying mean random effect terms (${\mathbf {b}}$). We form the joint posterior distribution over both the model parameters and auxiliary variables, given the observed data:

$$\begin{aligned} \pi ({\varvec{\theta }}, {\varvec{\phi }}| {\mathbf {B}}, {\mathbf {D}})\propto & {} f({\mathbf {B}}, {\mathbf {D}}, {\varvec{\phi }}| {\varvec{\theta }}) p({\varvec{\theta }}), \end{aligned}$$

where $f({\mathbf {D}}, {\mathbf {B}}, {\varvec{\phi }}| {\varvec{\theta }})$ denotes the joint likelihood of the observed data and associated auxiliary variables; and $p({\varvec{\theta }})$ the corresponding priors for the parameters (as defined in Sect. 3.3). The likelihood can be decomposed into the separate likelihood components described in Sects. 3.2.1–3.2.4. In particular, we can decompose the joint likelihood written as a product over each individual as follows:

$$\begin{aligned} f({\mathbf {B}}, {\mathbf {D}}, {\varvec{\phi }}| {\varvec{\theta }})= & {} \prod _{i=1}^I \underbrace{f({\mathbf {B}}_i | {\varvec{\theta }}, {\varvec{\phi }}) f({\mathbf {D}}_i | {\varvec{\theta }}, {\varvec{\phi }})}_{\text{ observation } \text{ process }} \times \underbrace{f(S_i | p_S) f(b_i | \sigma ^2_b) f(\tau _i | {\mathbf {C}}_i) f({\mathbf {C}}_i | \lambda _i)}_{\text{ system } \text{ process }} , \end{aligned}$$

where $\lambda _i$ is a deterministic function of $\zeta$, $\xi$ and $b_i$—see Eq. (8); and noting that the data (and auxiliary variables) are assumed to be conditionally independent given the parameters for each individual. The first two components of the joint likelihood correspond to the observation processes for the GALAD scores and diagnosis output given the true system states and model parameters (described in Sects. 3.2.1 and 3.2.4); the latter four components correspond to the underlying (unobserved) system process (described in Sects. 3.2.2 and 3.2.3).

Inference is carried out using Markov chain Monte Carlo (MCMC), obtaining a sample from the joint posterior distribution over the parameters and unknown system states (or auxiliary variables) given the observed data. To obtain a sample of the parameter values from the posterior distribution of only the parameters given the data, $\pi ({\varvec{\theta }}|{\mathbf {B}},{\mathbf {D}})$, (i.e. integrating out the auxiliary variables), we simply consider the simulated parameter values within the MCMC algorithm ignoring the associated simulated auxiliary variables. However, this Bayesian approach also provides immediate estimation of the associated auxiliary variables (or system states) by considering the associated simulated values within the MCMC algorithm, i.e. we also obtain a sample from the posterior distribution of the auxiliary variables. To conduct the MCMC simulations, we use the JAGS software (version 4.3.0) [25] and the R (version 3.5.0) [27] package rjags (version 4-6) [26]. The code for the JAGS model is available as supplementary material.

### Posterior predictive inference

The application to the detection of HCC using this model is based on the posterior predictive probability of the variable $C_{ij}=1$ for new observations of patients, as they indicate the probability of having developed HCC by time $t_{ij}$. Thus to assess this we consider this for the test dataset. Prediction by means of the GALAD score or serum biomarker will only be useful if this is before a diagnosis is made by other means. Therefore, within our assessment of our method for predicting the development of HCC we remove the diagnosis part of the prediction model (Eq. (10), nodes in orange in Fig. 1). Furthermore, patients who do not develop a change in their biomarker trend when developing HCC (i.e. sub-population 2) will not be distinguishable in prediction from those who do not develop HCC. To avoid competition of the parameter $S_i$ and the parameter $C_{ij}$ to model the lack of change of trend, we remove this mixture component and associated terms from the prediction model (Equation (3), nodes in red in Fig. 1). The variables to be predicted are not the GALAD scores (or biomarker levels) but the hidden states in the middle of the hierarchical model, i.e. the $C_{ij}$ values corresponding to whether or not an individual has HCC or not. Analytically expressing the posterior predictive distribution of the variable $C_{ij}$ is not feasible, therefore we use a computational approach fitting the model using the posterior distributions of the parameters for the training data as informative prior distributions for the parameters for the test data. As the information we have about the posterior distribution for the training data is a set of simulations, we approximate these distributions, checking that they fit the mean, variance and general form of the simulations. In particular, we use gamma distributions for $\sigma$ and $\sigma _b$, a normal distribution for $\beta$ and, due to the observed correlation of the simulations, a multivariate normal distribution for $(\nu , \zeta , \xi )$.

## Results

In this section we apply our proposed methodology to our motivating dataset of patients with cirrhosis being screened for HCC from the Ogaki Municipal Hospital, Japan, first estimating the parameters of the model using the training part of the dataset and then estimating the probability of having developed HCC at each observation for the test dataset. We evaluate the detection of HCC by our model in terms of sensitivity, specificity and timeliness (elapsed time between the model detection and the clinical diagnosis of HCC) and compare it with the established use of a static cut-point over the GALAD score.

### Estimation for the training data

We fitted the proposed model to the training dataset. Hyper-parameters were chosen to express vague prior beliefs of the parameters.

12 $$\begin{aligned} \beta&\sim {{\,\mathrm{U}\,}}(0, 0.1),&\nu&\sim {{\,\mathrm{{\mathcal {N}}}\,}}(0, 1000),&\delta&\sim {{\,\mathrm{U}\,}}(0, 0.15), \nonumber \\ \sigma&\sim {{\,\mathrm{U}\,}}(0, 100),&\zeta&\sim {{\,\mathrm{U}\,}}(-60, 0),&p_s&\sim {{\,\mathrm{Beta}\,}}(0.5, 0.5), \nonumber \\ \sigma _b&\sim {{\,\mathrm{U}\,}}(0, 100),&\xi&\sim {{\,\mathrm{U}\,}}(-1, 5),&\end{aligned}$$

The simulations were run on an Intel®Xeon®CPU E7-4830 v2 at 2.20 GHz and 64-bit Scientific Linux 7.4, part of a computer cluster, but the process was not parallelized, so only one core was used at a time. Two chains were run, each with 1000 non-Markovian adaptation iterations and 45,000 MCMC iterations, from which the first 7500 were discarded as burn-in. The convergence of the chains was checked by the Gelman-Rubin statistic [13] and by direct inspection of the plotted chains. The estimation of the parameters for the training data took 41.8 h. Mean values and 5th and 95th quantiles of the simulations for the posterior distributions of the parameters are shown in Table 2.

**Table 2:** Mean and quantiles 0.05 and 0.95 of the simulations of the estimated posterior distributions for the parameters of the model

	$\beta$	$\sigma$	$\sigma _b$	$\nu$	$\zeta$	$\xi$	$\delta$	$p_S$
$Q_{.05}$	0.00569	0.457	1.757	$-$3.219	$-$10.312	0.379	0.000236	0.564
Mean	0.00604	0.460	1.805	$-$3.218	$-$10.123	0.445	0.000312	0.656
$Q_{.95}$	0.00637	0.464	1.857	$-$3.144	$-$9.939	0.509	0.000398	0.741

The estimation of $\beta$ indicates that the mean GALAD level is estimated to increase around 0.006 points a day for those patients who have developed HCC (1.1 points over 6-months). The variability between patients, $\sigma _b$, is remarkably higher than within patients, $\sigma$, which indicates the importance to consider the personal baseline of the GALAD score in the model. The mean estimation of $\zeta$ is equivalent, by Eq. (8), to a mean intensity parameter $\lambda$ of 0.00004 for a person with average GALAD score level, which is equivalent to a probability of developing HCC of 0.0073 per 6 months (Eq. (6)). Following the estimation for $\xi$, the mean intensity parameter $\lambda$ for a patient with 2 points above and below the average GALAD score would be 0.00010 and 0.00002 respectively, with a corresponding probability of developing HCC in 6 months of 0.018 and 0.003, respectively. The estimated mean probability of being diagnosed in the trimester following the development of HCC according to Eq. (10) is 0.029, 0.11 in the following year and 0.30 in the following 3 years. The estimated probability $p_S$ of showing a change of trend on the GALAD scores for those patients who develop HCC is 0.66.

### Prediction for the test dataset

Predictions for the probability of having HCC were estimated for every observation within the last 1500 observed days for each patient. The prediction for each one of these observations was obtained by running the prediction model with informative priors several times, one for each of those observations of each patient. The model was run each time on a small dataset comprising the observation for which the prediction is to be made and all previous (but not posterior) observations for that particular patient, emulating the way that data would have been available in the real-life screening. The estimation took a total of 6909 s for the 4717 predictions, 1.5 s per estimation on average, which would be a reasonable computational time in real-life practice, where only one estimation would be done per visit. The informative prior distributions for the parameters were based in the estimations of the parameters for the training data:

13 $$\begin{aligned} \beta&\sim {{\,\mathrm{{\mathcal {N}}}\,}}(0.00604, 0.000207^2),&\begin{pmatrix} \nu \\ \zeta \\ \xi \\ \end{pmatrix}&\sim {{\,\mathrm{{\mathcal {N}}}\,}}\left( \begin{pmatrix} -3.218\\ -10.123\\ 0.445\\ \end{pmatrix} , \begin{pmatrix} 523 &{} -35 &{} -44\\ -35 &{} 113 &{} 175\\ -44 &{} 175 &{} 908\\ \end{pmatrix}^{-1} \right) , \nonumber \\ \sigma&\sim {{\,\mathrm{Gamma}\,}}(47{,}309, 102{,}754) \,,&\sigma _b&\sim {{\,\mathrm{Gamma}\,}}(3320, 1839) , \end{aligned}$$

with the parameters of the Gamma distributions expressed as shape and rate. The posterior probability of HCC can be interpreted as the risk of a person having developed a tumour that is associated with a change of trend of the GALAD score.

In order to evaluate the performance of the proposal as a detection tool for the development of HCC, measures of sensitivity, specificity and timeliness can be checked. Furthermore, receiver operating characteristic (ROC) curves can be built by varying the cut-points over the probability for declaring a detection. We shall compare the performance of this proposal with the use of a fixed threshold on the GALAD scores (from now on, just ‘threshold’ for simplicity), where a detection is declared whenever it is surpassed. The measures used have to consider the longitudinal nature of this problem. In that way, we consider the sensitivity per patient (proportion of detected patients over all patients diagnosed with HCC) and two different specificities; per patient (proportion of patients never detected over all patients not diagnosed with HCC) and per observation (proportions of non-detected observations belonging to patients not diagnosed over all the observations belonging to patients not diagnosed with HCC). We will also measure the timeliness of each method taken to be the mean distance between the diagnosis of HCC and the first time that a method consistently detects every observation from that one on (e.g. a value of 500 for the timeliness of one patient implies that the method detected that patient 500 days before being clinically diagnosed and that patient was also detected on every following observation).

**Fig. 2:**
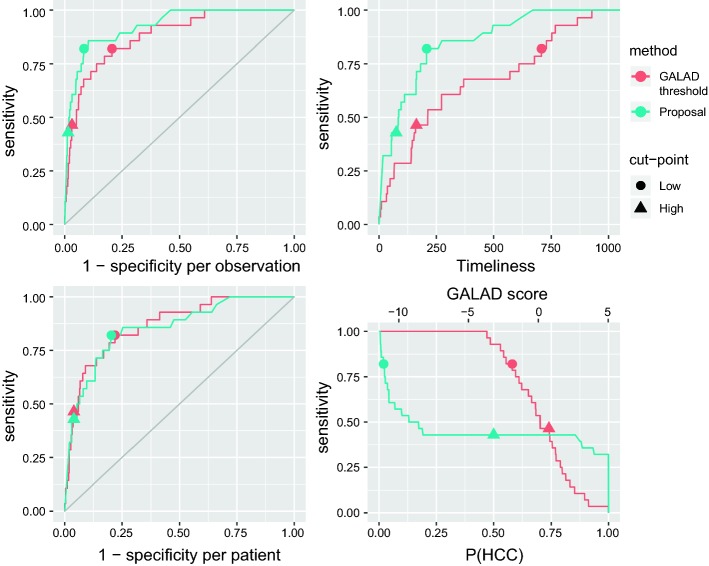
ROC curves using specificity per observation and per patient, sensitivity against timeliness and sensitivity against cut-point for the proposal (blue) and the threshold on the GALAD (red). Triangles (high) indicate 0.50 probability and 0.74 GALAD score cut-points. Circles (low) indicate 0.02 probability and $-1.87$ GALAD score cut-points (optimums) (colour figure online)

In addition to comparing the ROC curves (for both specificity per patient and per observation), we also provide curves of sensitivity versus timeliness and sensitivity versus the cut-point selected in Fig. 2.

**Fig. 3:**
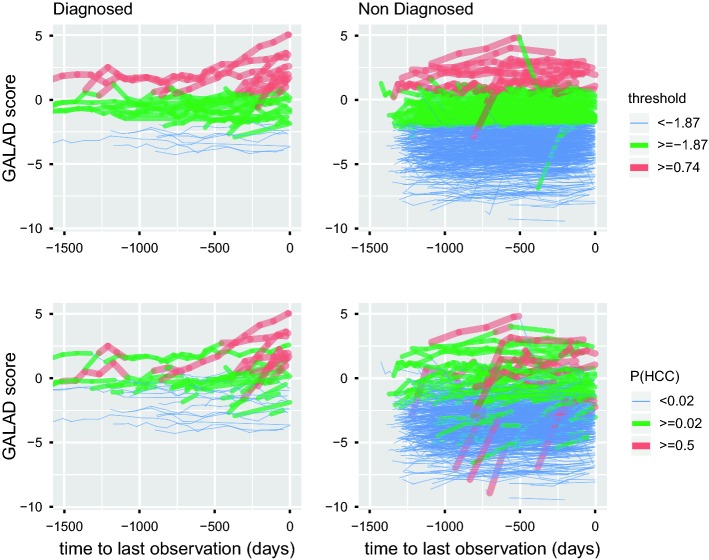
GALAD scores for the test dataset segmented by diagnosed and non-diagnosed with HCC. The top panels correspond to the static threshold method and the bottom panels correspond to the proposal. In red, observations that each method detects using the 0.50 probability and 0.74 GALAD score cut-points. In green and red, observations that each method detects using the 0.02 probability and $-1.87$ GALAD score cut-points (optimums). *P*(*HCC*) probability of HCC estimated by the new proposal (colour figure online)

In these curves we compare two different options for a cut-point for each method to further illustrate their performance. An obvious cut-point for our proposal is 0.5 probability of HCC as, when this cut-point is surpassed, our model estimates that the patient is more likely to have HCC than not. We compare this with a threshold of 0.74 over the GALAD which offers the same sensitivity per patient. Another option is to consider the cut-point or threshold that maximizes the sum of sensitivity and specificity per patient. In this way, a cut-point of 0.02 for the probability of HCC and a threshold for the GALAD score of $-1.87$ are the optimums for both sensitivity and specificity per patient. The optimum for our test dataset for the threshold is close to a previous optimum estimated over the same (whole) cohort [3] of $-1.95$. Sensitivity, specificity and timeliness for these two thresholds are very similar so we only show them for the optimum of our analysis for simplicity. Measures for the two pairs of cut-points are represented in Fig. 2 and shown in Table 3. Graphs of the detection for these cut-points for all the patients in the test dataset, segregated by diagnosed or non-diagnosed are shown in Fig. 3.

**Table 3:** Estimated measures of sensitivity (Sensit.), specificity per patient (Spec. P.), specificity per observation (Spec. O.) and timeliness for two different pairs of cut-points for our proposal and the threshold method

Method	Cut-point	Sensit.	Spec. P.	Spec. O.	Timeliness
Proposal	0.50	0.429	0.960	0.985	75
GALAD	0.74	0.464	0.960	0.968	163
Proposal	0.02	0.821	0.796	0.916	208
GALAD	$-$ 1.87	0.821	0.781	0.794	708

The first two have been matched by same specificity per patient. The last two are the optimums for the sum of sensitivity and specificity per patient

The ROC curve with specificity per patient (Fig. 2, bottom left panel) suggests similar performance of our approach when compared to the single threshold method, with area under the ROC curve (AUROC) for our proposal of 0.854 and for the threshold approach, 0.867. On the other hand, the ROC curve considering specificity per observation (Fig. 2, top left panel) shows consistently better performance for our proposal, with an AUROC of 0.926 compared to 0.880 for the threshold approach. In general, the sensitivity and specificity per patient of the two methods is comparable, but the specificity per observation (relevant for real time decision making) is consistently better for our proposed approach. The mean timeliness (Fig. 2, top right panel) is notably higher for the threshold, but the performance of the model over the longitudinal data depicted in Fig. 3 shows how this detection seems a significant period of time before diagnosis and sometimes reaches back a number of years prior to formal diagnosis. This suggests that the threshold may not be indicating the development of HCC but alternatively may identify individuals at particularly high risk of developing HCC in the future. Also, one can observe that the quantity of false positives per observation of our proposal seems to be lower and with less continuity over time, several times being just one isolated observation that shows a notorious increase in the level from the previous one.

## Discussion and conclusions

The early detection of many tumour types is a major clinical challenge across the world. Most tumours lie within the body thus are not visible and most are asymptomatic at least until they reach an advanced stage when treatment options become increasingly challenging. Thresholds for specific circulating blood biomarkers have been used to try to improve the sensitivity and timeliness of the detection for several types of cancer such as ovarian, prostate or HCC in a non-invasive and low costs manner, but with limited success [31, 35]. Actually, the longitudinal nature of the data when developing or evaluating these methods has generally not been taken into account, but instead only interpretation of a single measurement (possibly repeating the process over time) per patient has been used to aid in the discrimination of tumour development. Our evaluation of the performance of a simple threshold for the GALAD score suggests that this threshold could better be interpreted as an indicator of which patients are at higher risk of developing HCC in the long run than as an actual indicator of when a patient develops HCC.

In our new approach we address these issues by directly modelling the longitudinal data of the patients. Several authors have proposed methods which do consider the longitudinal nature of the data [2, 23, 36] but typically they consider a retrospective approach and focus on identifying the date of diagnosis for estimating the onset of disease. In this work we concentrate on the date of development of the tumour using the GALAD score, which is usually several months before a diagnosis by imaging or biopsy is able to detect the cancer. We propose a model based on current clinical understanding with a change-point component following a similar philosophy as other methods applied on ovarian cancer or HCC [22, 30, 34], while also dealing with important additional aspects of the nature of the data. This includes the use of an individual’s underlying mean GALAD score to be an associated risk factor for developing HCC, which was also identified independently using our investigation of the (traditional) threshold approach. Indeed, the strictly positive estimation of the parameter $\zeta$ indicates that the change of the risk of developing HCC for a patient regarding their baseline level is notable, more than doubling or halving the probability of developing HCC per half-year with a change of 2 points on the baseline GALAD score. Therefore taking account of this observed association between an individual’s baseline biomarker values and future HCC risk acts to strengthen the likeliness of tumour detection using this method.

An additional issue that our model directly addresses by using a HMM-type model is the existence of false negatives within the data, which are present due to the fact that imaging based diagnosis is only feasible months after the tumours initially develop. Ignoring these false negatives will lead to biased estimates of the model parameters, which could also have significant implications for predictive inference. The HMM components are able to address this issue by directly imputing the true HCC state of an individual within the MCMC algorithm—we note that the states are partially known in our case without error, in that a positive diagnosis implies that the true HCC state is known to be positive without error. In addition we directly model the time of onset of HCC without making it conditionally dependent on the diagnosis date, in contrast to previous works which typically model the onset by specifying prior distributions on the elapsed time between the onset and the diagnosis date. This previous approach leads to issues when we have no diagnosis within the test dataset (as it is the case in a real surveillance problem). One could evaluate such models with the test dataset where the time of diagnosis matches the last observation of the patient, but applying this prediction procedure to real data would necessarily diminish the capability of the model to detect the cancer on its first stages, as the time between the development of the tumour and the last observation available for that patient would be expected to be higher. In contrast our proposed prediction model does not use the information of the diagnosis date and therefore is suited to deal with real-time acquired data. Furthermore, we show that although complex to devise, this MCMC algorithm based approach is feasible to run from a data processing perspective within a healthcare system with individual sample estimation being possible in under 2 s.

The evaluation of the performance of longitudinal disease detection methods can present several problems, as is the ambiguity of the definition of specificity. The use of two different definitions of specificity—per patient and per observation—allows us to explore not only how many people would be given a false positive but also how many times that would happen over time. Another problem in evaluating these longitudinal methods is the correct separation of cases and controls of the prediction dataset. We have minimized the presence of potentially spurious controls (patients who have developed a tumour but it has not been diagnosed yet) by not considering the last 2 years of observations of the non-diagnosed patients of the test dataset. This, or other approaches, such as doing a follow-up of the diagnosis state of the patients during several years afterwards is necessary to avoid biased estimation of the specificity.

Another issue with basing cancer detection upon changes of serum biomarkers is the fact that some patients who develop HCC do not show a change of trend in their GALAD scores. They therefore will not be detected via the analysis of their biomarker data. In the present study, we have estimated that only around $66\%$ of the patients developing HCC show a change of trend, when using a composite score based upon three biomarkers. This proportion was estimated to be $81\%$ for Ovarian cancer detection based on the change of trend of the antigen CA125 [30] when combining the information of a previous study [18] (by means of an informative prior distribution) with the analysis of the data of a United Kingdom screening cohort [16]. Another study with data from the UK Collaborative Trial of Ovarian Cancer Screening (UKCTOCS), also using a similar prior distribution from the same previous information, estimated this probability to be $89\%$ for CA125 and $84\%$ for both Glycodelin and Human Epididymis Protein 4, but much lower (20–50%) for other selected biomarkers. For HCC it was found in a Taiwanese cohort that, when using an low absolute cut-point of AFP (9 ng/ml), over $30\%$ of HCCs will not be detected [7], which is consistent with the data from another cohort in the United Kingdom [4]. In general, this lack of change of the biomarker is a limitation that inevitably prevents the detection of some patients with any tumour type, but is particularly relevant for HCC surveillance. It is perhaps disappointing that when assessing changes in trends the inclusion of multiple biomarkers in the GALAD score does not improve this rate more significantly. It is notable the improvement that multiple biomarkers made to HCC detection sensitivity when comparing GALAD alone and AFP [17]. However, the detection of a change-point for at least one of several biomarkers could improve this situation [34].

This study uses a dataset from Japan, a country with a leading reputation in HCC detection and management [19]. Further work is required to validate this approach in other international cohorts. Given that, in general, tumours in this Japanese cohort were diagnosed at early stage (median maximum diameter 1.8 cm at diagnosis) then, conditional on those individuals who do develop a tumour, there is a potential drawback to a diagnostic technique such as the (static) GALAD threshold approach that provided repeated positive indications significantly before diagnosis—to the extent that many of these are likely to be prior to the establishment of a tumour. Consequently this could mean repeated futile attempts at diagnostic based imaging while no tumour has yet developed (so that there is a false positive screening indicator) or while the tumour is not of diagnosable size. Further, given the observed individual heterogeneity of the mean baseline GALAD scores, this may lead to repeated attempts of diagnostic tests of individuals with higher baseline scores due to natural variability, as opposed to a tumour present. Alternatively the proposed dynamic HMM approach using the complete patient longitudinal data has additional benefits over the traditional threshold based detection by both (i) using the change in GALAD scores which consequently removes the dependence on the individual mean baseline; and (ii) providing realistic (shorter) times between biomarker based indication and early stage diagnosis.

Current active research focuses on extending the proposed approach further by modelling several blood biomarkers simultaneously (AFP, AFP-L3 and DCP) [34], taking into account that they can evolve differently over time and with the onset of HCC. We are also exploring other ways of modelling the time from the HCC onset to the diagnosis, where an increasing instead of constant hazard is considered. Further we are investigating the inclusion of additional covariates such as age, gender, aetiology of the cirrhosis and time-varying health indicators in order to improve the predictive performance of the model.

In conclusion, in this paper we have presented a method for the analysis of longitudinal tumour biomarker data which permits the detection of the moment when a change in its trend occurs. This method is able to deal with real data both in the training of the parameters and the surveillance phases and could potentially be implemented within healthcare systems in the longer term.
